# Two Unrelated Families With Noncoding Duplications Upstream of MSX2 Refine the Critical Regulatory Region Likely Involved in Cranial Bone Development and a Cleidocranial Dysplasia‐Like Phenotype

**DOI:** 10.1155/humu/1258291

**Published:** 2026-08-17

**Authors:** Mamiko Yamada, Mark Cleghorn, Prabhakara Krishnamurthy, Tegan French, David Francis, Kenjiro Kosaki, Tiong Yang Tan

**Affiliations:** ^1^ Victorian Clinical Genetics Services, Parkville, Australia, vcgs.org.au; ^2^ Center of Medical Genetics, Keio University School of Medicine, Tokyo, Japan, keio.ac.jp; ^3^ Murdoch Children′s Research Institute, Parkville, Australia; ^4^ Department of Paediatrics, University of Melbourne, Melbourne, Australia, unimelb.edu.au

**Keywords:** cleidocranial dysplasia, *MSX2*, upstream duplication

## Abstract

Cleidocranial dysplasia (CCD) is a genetic disorder characterized by delayed cranial suture closure, hypoplastic clavicles, and dental anomalies, with varying severity. Most cases are linked to *RUNX2* variants; however, rare CCD‐like phenotypes can arise from other genetic alterations, including variants in *MSX2*, a critical skeletal development gene. Here, we describe two patients with persistent anterior fontanelles and microduplications upstream of *MSX2*, a region previously associated with CCD‐like phenotypes. Microarray analysis revealed narrower duplication ranges in these patients than in two earlier reported cases. To elucidate the underlying mechanisms, we performed in silico analyses using publicly available datasets. Epigenetic characterization of the minimal overlapping duplicated region (chr5:173848716–173888281) identified two transcriptionally active subregions showing high chromatin accessibility in osteoblasts. These findings suggest a regulatory role in osteogenic differentiation. Differential chromatin accessibility analysis using ATAC‐seq data demonstrated preferential accessibility in osteoblasts over chondrocytes in both human and mouse models, further implicating this region in bone development. The duplicated region was fully contained within a single topologically associating domain (TAD), suggesting that intra‐TAD duplications may disrupt spatial and temporal *MSX2* regulation, contributing to the CCD‐like phenotype. Phenotypic variations, such as synpolydactyly in a previously reported patient, likely arise from additional regulatory elements outside the minimal overlapping region. Our findings establish a causal link between *MSX2* upstream duplications and CCD‐like phenotypes, emphasizing the role of epigenetic mechanisms and TAD structures in regulating skeletal development. Further studies, including long‐read sequencing and ChIP‐seq, are needed to clarify the precise regulatory pathways involved.

## 1. Introduction

Cleidocranial dysplasia (CCD) is a genetic disorder characterized by a triad of delayed cranial suture closure, hypoplastic or aplastic clavicles, and dental anomalies [1]. The severity of CCD varies widely among individuals, presenting as a spectrum ranging from classic CCD to milder forms or isolated dental anomalies without other skeletal features [2].

Most CCD cases are caused by heterozygous variants in *RUNX2* (OMIM: 600211), a master transcription factor crucial for skeletal development [3]. Rarely, CCD‐like phenotypes arise from variants in other genes, such as *CBFB*, a downstream target of *RUNX2* (CBFB‐related CCD; OMIM: 620099) [4], or from chromosome 16q22 deletions (OMIM: 614541) [5]. Additionally, mutations in genes associated with RUNX2 function, including *CTSK* (pycnodysostosis) [6], *FIG4* (Yunis–Varon syndrome) [7], and *LMNA* (mandibuloacral dysplasia) [8], can produce overlapping features such as large fontanelles and clavicular hypoplasia, alongside unique phenotypic traits.

Like *RUNX2*, *MSX2* is critical for skeletal development, particularly in calvarial formation. Variants in *MSX2* result in distinct phenotypes depending on the nature of the mutation. Gain‐of‐function variants, such as specific missense mutations, cause Boston‐type craniosynostosis, characterized by premature cranial suture closure. Conversely, loss‐of‐function variants are linked to delayed fontanelle closure, hypoplastic clavicles, and parietal foramina, collectively termed parietal foramina with CCD (OMIM: 168550) [9]. These findings underscore the diverse roles of *MSX2* in cranial and skeletal development.

Previously, two patients with microduplications upstream of *MSX2* were reported to exhibit CCD‐like features [10]. Both displayed classic CCD traits, including absent clavicles and persistent anterior fontanelles, but one also showed atypical findings, such as synpolydactyly and postaxial polydactyly. The authors proposed that regulatory elements within the duplicated region disrupted MSX2 expression, affecting calvarial and clavicular development. This hypothesis was supported by evidence of high evolutionary conservation within the duplicated segment.

Conserved noncoding elements (CNEs) upstream of developmental genes often play critical roles in dynamically regulating spatial and temporal gene expression [11, 12]. Duplications of genomic regions containing CNEs can disrupt enhancer activity, altering the expression of key developmental regulators. Similar mechanisms have been observed in genes such as *SHH* (Sonic Hedgehog) [13], *IHH* [14], *BMP2* [15], and *SOX9* [16]. However, since the initial *MSX2* duplication cases, no additional patients with similar duplications have been reported, leaving the precise mechanisms underlying the CCD‐like phenotype unclear.

In this study, we describe two new patients with persistent anterior fontanelles and microduplications upstream of *MSX2*. The duplicated regions in these patients are narrower than those in previously reported cases. In silico epigenetic analysis of the minimally overlapping duplicated region provides additional insights into how *MSX2* upstream duplications contribute to CCD‐like phenotypes.

## 2. Clinical Report

### 2.1. Patient 1

**Figure 1:**
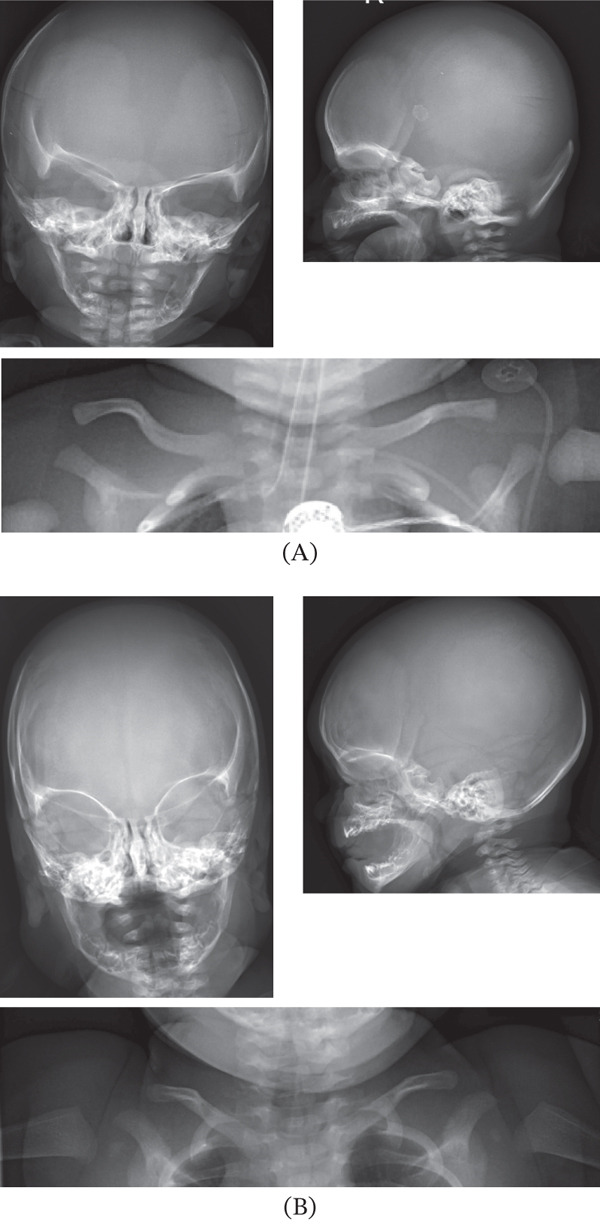
Skull radiographs. (A) Patient 1 (Day 8) (left: AP skull, right: lateral skull, bottom: clavicles): note the wide anterior fontanelle with widely patent sagittal and metopic sutures, with normal clavicles. (B) Patient 2 (2 months) (left: AP skull, right: lateral skull, bottom: clavicles): note the poorly ossified skull, wide anterior fontanelle with wide intraparietal sutures, but normal clavicles.

The first patient was a 4‐year‐old boy born at 39 weeks of gestation to healthy unrelated parents with no significant family medical history. His birth weight was 3110 g (−0.57 SD), his birth length was 49 cm (−0.55 SD), and his birth head circumference was 33.6 cm (−0.83 SD). He had a wide anterior fontanelle with splayed sagittal and metopic sutures (Figure 1A), bilateral clinodactyly of the fifth fingers and normal clavicles to palpation. Neither parent had any similar findings and had no history of delayed fontanelle closure. At the age of 3 years and 5 months, his weight was 15.2 kg (0.08 SD), his height was 101 cm (0.87 SD), and his head circumference was 50 cm (0.14 SD). His anterior fontanelle was persistently open. His development was age‐appropriate.

### 2.2. Patient 2

The second patient was a 7‐year‐old girl born at 37 weeks of gestation to unrelated parents. Her birth weight was 2615 g (−1.42 SD), her birth length was 51.5 cm (+1.26 SD), and her birth head circumference was 30.5 cm (−3.0 SD). She was referred to genetics in the first month of life due to poorly ossified parietal bones with continuity between her anterior and posterior fontanelles and open intraparietal sutures bilaterally (Figure 1B) but normal clavicles and limbs. Her mother had also been referred to genetics as a neonate because of a poorly ossified skull with large anterior and posterior fontanelles, which closed at the age of 18 months, bilateral short and broad distal phalanges of the thumbs, and normal clavicles. Patient 2′s mother was clinically followed and demonstrated normal dental eruption, growth, and development. Patient 2′s two brothers had normal size anterior fontanelles. Patient 2′s dental eruption was normal, with normal dentition and her anterior fontanelle closed by age 2.5 years.

## 3. Genetic Investigation

This study was conducted as part of standard clinical care and informed consent was obtained from the patients′ parents for molecular studies. The analysis was performed using genomic DNA extracted from peripheral whole blood.

### 3.1. Chromosomal Microarray Analysis

Chromosome microarray was performed using the Infinium ImmunoArray‐24 v2.0 BeadChip Kit for Patient 1 and Infinium CoreExome‐24 v1.1 BeadChip Kit for Patient 2 (Illumina, Infinium CoreExome‐24 v.1.1). Microarray slides were scanned using the iScan System (Illumina, San Diego).

In Patient 1, a 1.48‐Mb duplication of chromosome 5q35.1q35.2 was detected: Minimum genomic coordinates were arr[GRCh37]5q35.1q35.2(172,412,553_173,888,281) ×3 mat and maximum genomic coordinates were arr[GRCh37]5q35.1q35.2(172,409,822_173,905,800) × 3 mat. His mother was calculated to be approximately 40% mosaic for the same duplication. The level of mosaicism (variant allele fraction‐VAF) was calculated using previously published VAFs for Illumina SNP microarrays [17] (see microarray data, Figure S1). It is well‐recognized that the level of mosaicism can vary significantly across different tissues. The 40% mosaicism observed in peripheral blood might not represent the proportion in other embryonic lineages, which may explain the lack of a discernible clinical phenotype in the mother.

In Patient 2, an approximately 0.6‐Mb duplication of chromosome 5q35.2 was detected: Minimum genomic coordinates were arr[GRCh37]5q35.2(173,416,324_174,025,065) × 3 mat and maximum genomic coordinates were arr[GRCh37]5 q35.2(173,412,816_174,036,190) × 3 mat. Her mother also had the same duplication. Her unaffected brothers were found to not have inherited the duplication.

**Figure 2:**
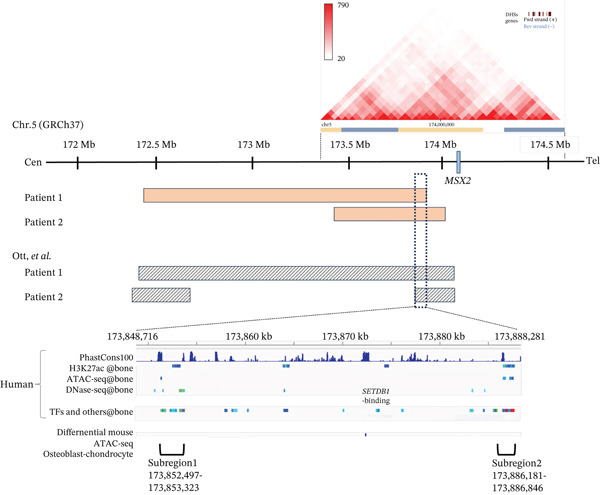
Minimal overlap region of duplication and epigenetic modifications. Top panel: Hi‐C interaction heatmap of the MSX2 genomic region at 40 kb resolution was generated using the 3D Genome Browser (http://3dgenome.org). Chromosomal positions of TADs are indicated by yellow and cerulean blue bars. The minimal overlapping duplication region and the MSX2 coding region are both fully contained within a single TAD spanning chr5:173,880,000–174,240,000 (highlighted in yellow). Hi‐C data were reprocessed from embryonic stem cell data (H1‐ESC cells). Middle panel: The duplicated regions of the two patients in this study (Patient 1 and Patient 2) and two previously reported by Otto et al. are shown. The minimal overlapping duplication region across all four patients is highlighted by a dotted rectangle. Bottom panel: Epigenetic characterization of the minimal overlapping duplication region across four patients. PhastCons 100: evolutionary conservation among 100 vertebrates; H3K27ac @Bone: signal peaks of 278 H3K27 acetylation experiments using bone tissues or cells; ATAC‐Seq @Bone: signal peaks from 399 ATAC‐seq experiments in bone tissues or cells; DNase‐seq @Bone: signal peaks from 32 DNase‐seq experiments; TF and others @Bone: signal peaks from 865 ChIP‐seq experiments targeting transcription factors in bone tissues or cells; Differential mouse ATAC‐seq (osteoblast vs. chondrocyte): mouse ATAC‐seq positive regions with significantly higher accessibility in osteoblasts compared with chondrocytes (see main text).

The duplication ranges of the four patients—two from a previous publication [10] and two from the present study—were mapped onto the human reference genome (GRCh37) of chromosome 5 (Figure 2, middle panel). The minimal overlapping region among the duplicated segments across these four patients, all presenting with patency of the anterior fontanelle, spans approximately 40 kb: chr5(GRCh37):g.173848716–173888281.

### 3.2. Epigenetic Characterization of the Microduplication

According to the 3D Genome Browser (https://3dgenome.fsm.northwestern.edu) [18], which gives a comprehensive view of both regulatory landscape and 3D genome structure, the minimal overlap of duplication and the coding region of MSX2 were both completely included within a single topologically associating domain (TAD) [19] spanning from chr5:173,880,000 to 174,240,000 (Figure 2, top panel). TAD boundaries are known to facilitate enhancer‐promoter and enhancer‐enhancer interactions within TADs, suggesting that the identified duplicated region may play a spatially organized regulatory role in bone differentiation.

To identify regulatory elements potentially responsible for the CCD‐like phenotype within the overlapped region, we reprocessed publicly available ChIP‐seq, ATAC‐seq, and DNAse‐seq data integrated via the ChIP‐Atlas platform [20] (https://chip-atlas.org/). ChIP‐Atlas is a comprehensive and curated repository of publicly available epigenetic data, including ChIP‐seq studies. ChIP‐seq identifies DNA regions bound by transcription factors or histone modifications, such as H3K27ac, which marks active enhancers and promoters [21]. Similarly, ATAC‐seq [22] and DNase‐seq [23] detects open chromatin regions accessible to transposase digestion, reflecting active regulatory sites.

Using the Peak Browser function on the ChIP‐Atlas website, we detected “peaks,” representing short epigenomic regions enriched with epigenetic markers or factors compared with background levels [24], within the minimal overlapping duplication region (chr5:173848716–173888281, hg19) across four patients. Two subregions were identified as transcriptionally active in bone tissues: Subregion 1 (chr5:173852497–173853323) and Subregion 2 (chr5:173886181–173886846). Both subregions displayed open chromatin configurations, suggesting transcriptional activity in bone tissues (Figure 2, middle panel).

Differential chromatin accessibility analysis using publicly available ATAC‐seq datasets from mouse osteoblasts [25] (SRX11156877, SRX11156878, SRX11156879) and mouse chondrocytes (SRX11156874, SRX11156875, SRX11156876) showed that chr13:53765842‐53765970 in mouse (mm9) exhibited higher chromatin accessibility in osteoblasts than in chondrocytes, suggesting preferential transcriptional activity during osteogenic differentiation. This mouse genomic region maps to chr5:173848716–173888281 in human genome coordinate hg19 according to the liftOver function of the UCSC genome browser (Figure 2, bottom panel). This genomic region was highly conserved with high PhastCons score [26] and is inferred to play a critical role in the formation of bone. This region was flanked by a SETDB1‐binding site, which was detected in osteosarcoma‐derived ChIP‐seq data (SRX150537, GSM935458).

## 4. Discussion

We report two additional patients with CCD spectrum disorder and persistent anterior fontanelle, both carrying a microduplication upstream of the *MSX2* gene. This finding aligns with the previous report of two patients with similar duplications presenting comparable CCD spectrum phenotypes and further refines the critical region. The minimal overlapping region (chr5:173848716–173888281) including two subregions (chr5:173852497–173853323 and chr5:173886181–173886846) exhibit transcriptional activity and differential chromatin accessibility favoring osteoblasts. Our observation supports a causal relationship between upstream *MSX2* microduplications and the CCD spectrum phenotype, rather than a coincidental association. In silico interrogation of the 5q35 breakpoint region identified an AluSg4 element at the duplication boundary, in the absence of large segmental duplications. This genomic architecture is compatible with repeat‐mediated rearrangement mechanisms, including Alu‐driven nonallelic homologous recombination (NAHR). However, given the resolution limits of array‐based detection, the precise breakpoint positions remain defined within probe intervals, and the structural configuration of the duplication cannot be resolved. Accordingly, multiple scenarios remain plausible, including tandem duplication at the native locus, inverted duplication, or insertion at a distal genomic site. These structural uncertainties represent an important limitation and should be considered when interpreting potential effects on regulatory architecture and MSX2 expression. Interestingly, the minimal overlapping duplication region shared by the four patients is located within a single TAD, Figure 2, top part), as observed in a human ChIP‐seq experiment [18]. This observation aligns with the general notion that duplications confined within a single TAD are often more disruptive to gene regulation than duplications spanning TAD boundaries [27]. Such an intra‐TAD duplication could lead to either a simple dosage imbalance or a more complex three‐dimensional structural disruption involving this region. These disruptions may, in turn, have altered the spatial and temporal expression of the *MSX2* gene, ultimately affecting calvarial suture formation.

When comparing the phenotypes of the four individuals with a microduplication upstream of the *MSX2* gene, individual phenotypic differences were observed, despite the shared feature of delayed cranial suture closure. For example, one of the two patients described by Ott et al. (Patient 2) exhibited synpolydactyly [10], whereas the remaining three patients did not display this feature. There are two possible explanations for these phenotypic differences. The first is the possibility of an unrelated causal variant in a gene responsible for limb malformation. Although Otto et al. reported that no pathogenic variants were detected when they sequenced six genes associated with limb malformations, there are 155 limb malformation genes listed in Genomics England PanelApp currently (https://panelapp.genomicsengland.co.uk/panels/384/). Second, it is possible that the structural variant has disrupted one of the limb developmental pathways such as FGF, Wnt, and SHH signaling [28]. The regulatory elements involved in hand digit formation may reside within the duplicated region present only in Patient 2, potentially in regions such as chr5:174025065–174066198. Synpolydactyly, as a subset of syndactyly‐related birth defects, suggests abnormalities in apoptotic processes during digit formation. Given that *MSX2* acts as a downstream effector of *SHH* [29], it is plausible that the duplicated segment specific to patient P2 contains regulatory elements interacting with the SHH signaling pathway. Further studies are needed to identify the transcription factor(s) upstream of *MSX2* that may be responsible for hand patterning defects.

Furthermore, the patients described by Ott et al. were found to have clavicular agenesis. The genomic region chr5:174,036,190–174,066,198, located approximately 110 kb upstream of *MSX2*, may represent a distal regulatory element involved in clavicular development. Bioinformatic analysis using ChIP‐Atlas revealed enrichment of H3K27ac and increased chromatin accessibility (ATAC‐seq and DNase‐seq) at this locus in osteoblastic cell lines, including U2OS and SaOS‐LM1. Binding of EPAS1, a transcription factor implicated in skeletal and chondrocyte differentiation, was identified in this region. Binding of RUNX2, a master regulator of osteoblast differentiation, was also observed in the vicinity of this locus in ChIP‐Atlas datasets, although its peak positions did not completely overlap with regions of H3K27ac enrichment and chromatin accessibility (Figure S2). This discrepancy may reflect cell type–specific binding patterns or the presence of multiple regulatory elements within the region. Given the key role of *MSX2* intramembranous ossification, these epigenetic features are consistent with the possibility that this locus functions as a cis‐regulatory element of *MSX2*. However, whether this region directly regulates *MSX2* expression and contributes to the clavicular phenotype remains unclear. Structural variations or mutations affecting this locus may influence *MSX2* regulation, but further functional validation is required. In the present study, the presence of the duplication was successfully demonstrated in two patients using microarray analysis. A limitation of this study is in defining the structural nature of the duplication, whether it be direct or inverted, tandem or inserted elsewhere in the genome. This characterization would be helpful to elucidate how the regulatory region may have been affected and any subsequent functional changes to *MSX2* protein expression. Long‐read sequencing could potentially elucidate the primary structure of the duplication [30], whereas ChIP‐seq analysis might offer insights into its conformational structure. Additional genomic studies for further validation and analysis can form the basis for future research.

In conclusion, we report two patients with microduplication of the upstream noncoding region of *MSX2*. By comparison with two previously reported patients with overlapping microduplications, the critical region was refined to (chr5:173848716–173888281) including two subregions (chr5:173852497–173853323 and chr5:173886181–173886846), which exhibit transcriptional activity and differential chromatin accessibility favoring osteoblasts. We propose that the duplication of the putative RUNX2 binding elements located within the minimal overlapping region may play a biological role in driving the CCD‐like calvarial defect.

**Nomenclature**
CCD: cleidocranial dysplasia
SHH: Sonic Hedgehog
TAD: topologically associating domain

## Author Contributions

All authors contributed to the study conception and design. Material preparation and data collection were performed by P.K., D.F., T.F., and T.Y.T. Genetic analysis was performed by P.K. and D.F. The first draft of the manuscript was written by M.C. and M.Y. All authors commented and contributed to the final versions of the manuscript. M.Y. and M.C. equally contribution.

## Funding

This study was supported by The Japan Foundation for Pediatric Research (F23‐001); RDNow knowledge exchange scholarship (2023‐1484) awarded to Mamiko Yamada as part of a grant provided by the Royal Children’s Hospital Foundation.The Rare Disease Flagship acknowledges financial support from the Royal Children’s Hospital Foundation, the Murdoch Children’s Research Institute, The Harbig Foundation, The Fox Family Foundation, The Andrew and Geraldine Buxton Foundation and The Pierce Armstrong Foundation. The research conducted at the Murdoch Children’s Research Institute was supported by the Victorian Government′s Operational Infrastructure Support Program.

## Disclosure

All authors read and approved the final manuscript.

## Conflicts of Interest

The authors declare no conflicts of interest.

## Supporting Information

Additional supporting information can be found online in the Supporting Information section.

## Supporting information


**Supporting Information 1** Figure S1: SNP microarray profile in the 5q35 region of the mother of Patient 1. (a) The copy number gain (CNx3, shaded blue) of 40% proportion. (b) B‐allele (BAF) splitting confirming the 40% mosaicism. Red serrated lines indicate the expected nonmosaic copy number and B allele states. Minimum genomic coordinates for the mosaic duplication were arr[GRCh37]5q35.1q35.2(172,412,553_173,888,281) × 3.

**Supporting Information 2** Figure S2: Epigenetic landscape and transcription factor binding at the putative regulatory region upstream of *MSX2*. Integrative Genomics Viewer (IGV) snapshot of the genomic region chr5:174,036,190–174,066,198 (hg19), located approximately 110 kb upstream of MSX2. Tracks derived from ChIP‐Atlas show H3K27ac enrichment and chromatin accessibility signals (ATAC‐seq and DNase‐seq) in osteoblastic cell lines, including U2OS and MG‐63. Binding of EPAS1 is detected within this region. Binding of RUNX2 is observed in osteoblastic and mesenchymal datasets in the vicinity of this locus.

## Data Availability

The data that support the findings of this study are available from the corresponding author upon reasonable request.
